# Hepatitis E virus seroprevalence in South Africa: a multi-site study among HIV-negative and HIV-positive adults and age-stratified children (2–17 Years)

**DOI:** 10.1186/s12879-026-13905-3

**Published:** 2026-07-07

**Authors:** Tarun Saluja, Nigus Telele, Elizabeth Hellström, Essack Mitha, Maphoshane Nchabeleng, Rita Baiden, Naveena Aloysia D’Cor, Sridhar Vemula, Ju Yeon Park, Lei Yang, Jiyoung Lee, Deok Ryun Kim, Sunju Park, Sanet Aspinall, HuiRong Pan, J Wai-Kuo Shih, Julia A Lynch

**Affiliations:** 1 International Vaccine Institute, Seoul, Republic of Korea; 2 International Vaccine Institute, Europe Regional Office, Stockholm, Sweden; 3 Be Part Research, Mbekweni, Paarl, Western Cape South Africa; 4 Newtown Clinical Research Centre, Johannesburg, South Africa; 5 Mecru Clinical Research Unit, Department of Microbiological Pathology, National Health Laboratory Service, Sefako Makgatho Health Sciences University, Pretoria, South Africa; 6 Ardent Consulting (Pty) Ltd, Cape Town, South Africa; 7 Xiamen Innovax Biotech Co., Ltd., Xiamen, China

**Keywords:** HEV, Seroprevalence, South Africa, Multi-site, Adults, Children, HIV, Antibody titers

## Abstract

**Background:**

Hepatitis E virus (HEV) seroprevalence varies by age and geography. Data on HEV seroprevalence across age groups and among people living with HIV (PLWH) in South Africa is scarce.

**Methods:**

We conducted a prospective multi-site assessment of anti-HEV IgG seroprevalence on 859 South African participants enrolled at three clinical research centres including Newtown Clinical Research Centre in Johannesburg, Be Part Research in Mbekweni, Western Cape, and Mecru Clinical Research Unit in Garankuwa, Pretoria. Participants comprised adults aged 18–45 years (PLWH, *n* = 178 and HIV-negative, *n* = 232), and children aged 2–17 years (*n* = 449). Anti-HEV IgG serostatus and antibody titer were measured using a commercial ELISA kit and a WHO reference standard. Seroprevalence was assessed by site, age group, sex, and HIV status.

**Results:**

Overall anti-HEV IgG seroprevalence was 18.0% (95% CI: 15.6–20.8). Adults had the highest seroprevalence (27.3% among all adults; 29.2% among PLWH and 25.9% in HIV-negative adults), while adolescents aged 12–17 years had the lowest (6.9%), and young children aged 6–11 years and 2–5 years had 10.3% and 13.0%, respectively. Adults had significantly higher odds of seropositivity than children (aOR 2.8, 95% CI: 1.5–5.5, *p* = 0.002). A significant site-specific variation was also observed among healthy adults and adolescents: Newtown Clinical Research Centre (23.0% and 14.0%) and Be Part Research (34.5% and 7.3%) had higher seroprevalence compared with those from Mecru Clinical Research Unit (17.2% and 1.5%, *p* = 0.0499 and *0.0262*, respectively). Higher mean antibody titer observed in younger children aged 2–5 years (5.06 IU/mL), compared with adults (0.88 IU/mL among PLWH and 0.68 IU/mL among HIV-negative adults), and older children (2.02 IU/mL in those aged 6–11 years and 0.67 IU/mL in those aged 12–17 years).

**Conclusions:**

HEV seroprevalence in South Africa was highly heterogeneous, varying markedly by age group and study site. These findings highlight the need for strengthened, integrated HEV surveillance to better define transmission patterns and to inform evidence-based considerations for prevention of infection.

**Trial registration:**

ClinicalTrials.gov: NCT06306196; Registration date: 2024-02-18. South African National Clinical Trials Register (SANCTR): DOH-27-032024-8165; Registration Date 2024-03-04.

## Background

Hepatitis E virus (HEV), identified in the early 1980s, is one of the most common global causes of acute viral hepatitis [1], with an estimated 19.47 million cases of acute hepatitis E (AHE) globally in 2021 [2]. The highest global disease burden is observed in regions with limited access to clean drinking water, as fecal contamination of water sources remains a primary mode of transmission [3, 4]. HEV consists of four primary genotypes [1–4] that infect humans. Genotypes 1 and 2 are exclusively human pathogens and are primarily spread through the fecal-oral route, often triggering large-scale outbreaks in areas with inadequate sanitation [5]. On the other hand, genotypes 3 and 4 are zoonotic, linked to the consumption of undercooked meat or direct contact with infected animals, and are more commonly found in developed regions [6].

While HEV infection is often self-limiting in immunocompetent individuals, it can cause severe complications in vulnerable populations, including immunocompromised individuals and pregnant women [7, 8]. In people living with HIV (PLWH) with low CD4 + T-cell counts and impaired immune responses, HEV can persist, leading to chronic hepatitis and progressive liver fibrosis [9]. Studies indicated that HEV seroprevalence is higher among HIV-infected individuals compared to the general population [10, 11] and HEV-HIV co-infection may accelerate progression to liver cirrhosis, complicating clinical management [12].

In addition, children represent a vulnerable population affected by HEV though traditional serological tests may lack diagnostic precision, especially in areas with high endemicity [13, 14]. Although HEV infection is usually self-limiting in immunocompetent children, those with underlying liver diseases, or immunocompromised children, are at higher risk for severe, prolonged or recurrent infections [14, 15]. In the pediatric population, diagnosis can be difficult due to nonspecific symptoms, and the disease often remains underdiagnosed due to its overlap with other childhood illnesses [16]. Serological tests detecting anti-HEV IgM and IgG antibodies are used for diagnosis, but routine screening is not typically performed [17]. Importantly, HEV infection during pregnancy is also associated with substantially increased maternal morbidity and mortality [18, 19].

In South Africa, studies conducted in the 1990s reported HEV seroprevalence rates ranging from 2% to 10% [20, 21], but these early estimates were limited by small sample sizes and regional focus. However, recent studies have indicated that HEV is endemic in the country, and a high seroprevalence has been reported [20–24]. The studies suggest a mix of waterborne and zoonotic transmission, with increasing genetic diversity of the virus detected in pig products [24]. However, routine HEV screening is not a standard practice in South African healthcare, which results in significant underreporting and a lack of comprehensive surveillance data [20].

Recent studies indicate a rising burden of HEV infection, with seroprevalence rates increasing, though they vary considerably across different provinces. In the Western Cape, a seroprevalence of 27.9% has been reported, with higher rates observed among individuals over 30 years of age, and with pork and bacon/ham consumption suggested as a risk [22], suggesting zoonotic transmission may be a factor. Similarly, in the Free State, seroprevalence was significantly higher at 60.9%, highlighting substantial past exposure in certain populations [23]. These findings suggest that HEV is more prevalent than previously recognized, but its public health importance remains underestimated. Despite the increasing evidence of widespread HEV exposure in South Africa, data specific to high-risk populations, such as immunocompromised individuals and children, are still limited as most available studies have been conducted in adult or mixed-age populations without detailed age-specific estimates, highlighting an important gap in understanding early-life transmission dynamics.

Therefore, the objective of this study was to determine the seroprevalence of anti-HEV IgG antibodies among individuals aged 2–45 years enrolled in a vaccine trial across three clinical research sites in South Africa. In addition, the study aimed to compare seroprevalence across key demographic subgroups, including geographic location, age, sex, and HIV status, and to quantify anti-HEV IgG titers among seropositive participants using a WHO-standardized reference. We used anti-HEV IgG as it reflects past exposure and cumulative seroprevalence.

## Methods

### Study sites and population

Baseline blood samples were collected from participants enrolled from three clinical trial sites in South Africa participating in a Phase 2b, open-label study to evaluate the immunogenicity and safety of Hecolin^®^ in HIV positive/negative adult participants and children (ClinicalTrials.gov: NCT06306196; registration date: 2024-02-18 and South African National Clinical Trials Register (SANCTR): DOH-27-032024-8165; Registration Date 2024-03-04). They were stratified by age into four different cohorts: 18–45 years, 12–17 years, 6–11 years, and 2–5 years. The adult cohort was further stratified into HIV-positive and HIV-negative groups. The trial sites include Be Part Research (Mbekweni, Paarl in Western Cape), Newtown Clinical Research Centre (in Johannesburg), and Mecru Clinical Research Unit (in Garankuwa, Pretoria).

The Newtown Clinical Research Centre is a clinic-based research centre in urban Johannesburg. The Be Part Research site is located in Mbekweni, Drakenstein sub-district of peri-urban Cape Town. The Mecru Clinical Research Unit (MeCRU) is a university-based clinical trials site at Sefako Makgatho Health Sciences University (SMU) based in Garankuwa in peri-urban Pretoria. Participants were recruited for the trial from their communities through outreach activities, referrals from local health services, and direct engagement with past volunteers.

### Sample size

No formal sample size calculation was performed for this seroprevalence analysis, as this was an exploratory analysis conducted using all available baseline samples for enrolled participants prior to vaccination in the parent clinical trial (*N* = 860).

### Sample collection and processing

Approximately 3.5 mL blood sample was collected from each participant prior to vaccination using a Gold-Top serum separator tube (SST). The blood samples were centrifuged within one hour of collection at 1400 g for 10 min at the clinical sites and shipped refrigerated to Cytespace Africa Laboratories, a clinical laboratory in Pretoria, on the same day. Serum was separated, aliquoted, and stored at -70 °C until analysis at Cytespace Laboratories. Before enrolment, adult participants not on antiretroviral (ARV) therapy were tested for HIV at the respective clinical sites. Participants with a prior HIV diagnosis were required to be on stable antiretroviral (ARV) therapy, and newly diagnosed participants were required to have received ARV for at least four weeks prior to enrolment.

### HEV serological testing

Anti-HEV IgG was measured using the WANTAI HEV IgG ELISA kit (Beijing Wantai Biological Pharmacy Enterprise, Beijing, China) following the manufacturer’s instructions. Only anti-HEV IgG seropositive samples were further tested with a quantitative ELISA developed in-house using a WHO Reference Reagent for antibodies to hepatitis E virus, human serum (NIBSC code: 95/584) [25, 26].

Additionally, for HIV-positive participants, HIV viral load was measured using the GeneXpert platform with a detection limit of 40 copies/mL, while CD4% and absolute CD4 counts were determined using the BD FACSCanto II flow cytometer. All assays were conducted according to the manufacturer’s instructions and standard laboratory protocols to ensure precise and reliable results.

### Statistical analysis

All data were entered into an electronic data capture system, and analyses conducted using SAS 9.4 (SAS Institute, Cary, NC). Descriptive statistics were used to summarize demographic characteristics and anti-HEV IgG seroprevalence rates at baseline (before vaccination). Given the very small number of participants with equivocal/borderline anti-HEV IgG results, they were not excluded from analyses. Seroprevalence was compared across age groups, sex, geographic location of the clinical sites, and potential risk factors such as HIV status, viral load, and CD4 cell count. Categorical variables were analyzed using chi-square or Fisher’s exact tests, while continuous variables were assessed using t-tests or Mann-Whitney U tests for two groups, ANOVA or Kruskal–Wallis test for more than two groups as appropriate. Seroprevalence was estimated for each demographic subgroup such as sex, HIV status, and age group, and by study site, with 95% confidence intervals calculated using the Wilson score method. Crude odds ratios (ORs) were estimated using simple logistic regression for each variable individually. Adjusted odds ratios (aORs) were estimated using multivariable logistic regression models. Age group and study site were included as prespecified adjustment covariates based on the stratification factors used in the randomization scheme. For each analysis, the model included the variable of interest together with these adjustment covariates. When age group or site was the variable of interest, the corresponding variable was omitted from the adjustment set. No interaction terms were evaluated given the exploratory nature of the analysis and limited sample size. All statistical tests were two-sided with a significance level of 5%.

## Results

**Fig. 1:**
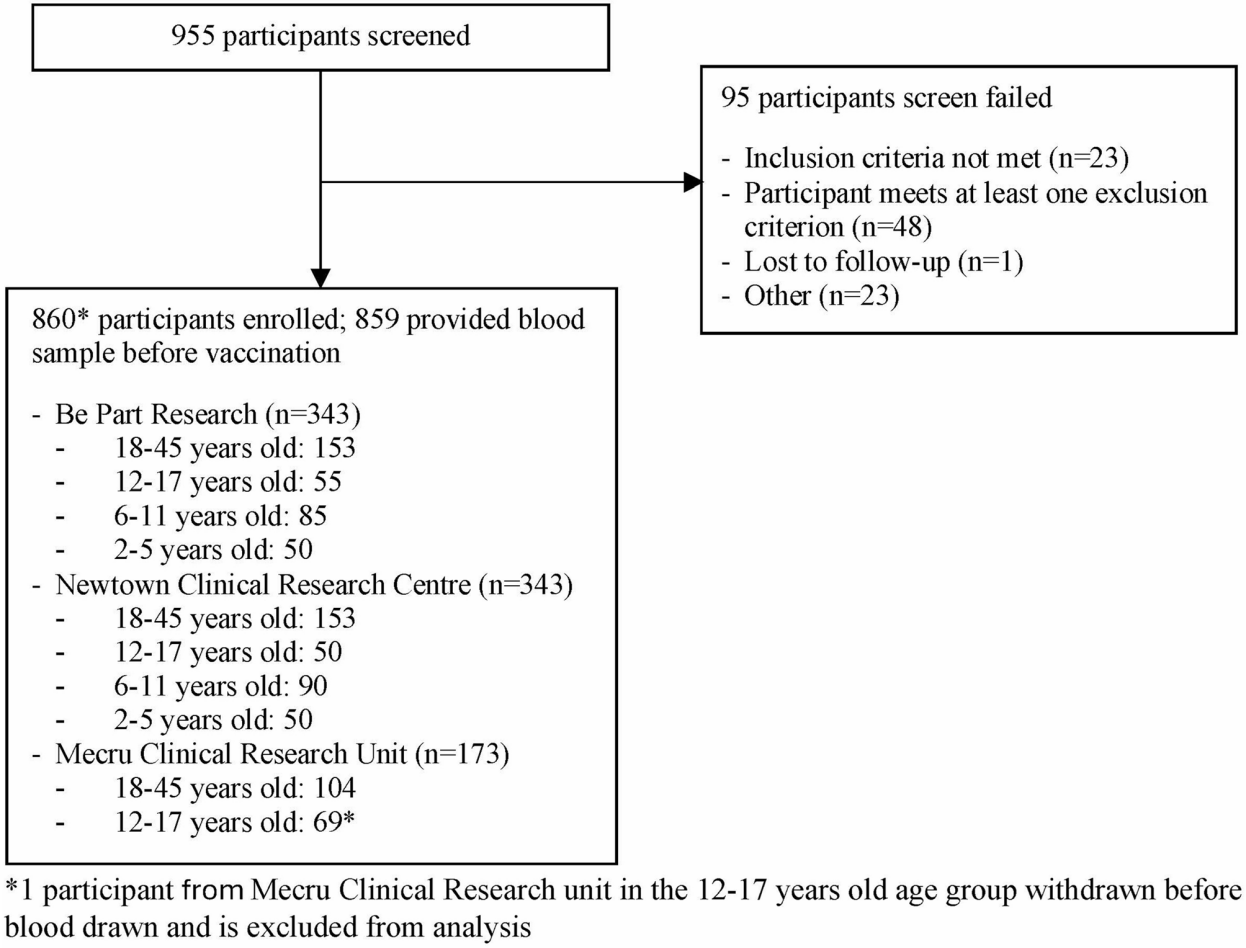
Flowchart illustrating participant enrolment, and inclusion in the final analyses

A total of 955 participants were screened for eligibility in the trial, of whom 95 did not meet the inclusion criteria, and 860 participants were enrolled across three sites in South Africa (Fig. 1). All participants provided baseline blood samples prior to vaccination, except one who withdrew before sample collection. This resulted in a total of 859 participants included in the analysis across the three sites: Be Part Research (Mbekweni in Western Cape) and Newtown Clinical Research Centre (in Johannesburg) each enrolled 343 participants, while Mecru Clinical Research Unit (Garankuwa in Pretoria) enrolled 173 participants.

### Demographic and clinical characteristics

The study population included adults living with HIV (PLWH, *n* = 178), HIV-negative adults (*n* = 232), and healthy participants from three pediatric cohorts (12–17 years, *n* = 174; 6–11 years, *n* = 175; 2–5 years, *n* = 100). Summaries of the demographic and clinical characteristics of participants stratified by HIV status and age group are presented below in Table 1.

**Table 1:** Demographic and clinical characteristics by age group and HIV status (*N* = 859)

Characteristics	Total (*n* = 859)	Adult PLWH*	HIV-negative adult	Children participants		
		18–45 years (*n* = 178)	18–45 years (*n* = 232)	12–17 years (*n* = 174)	6–11 years (*n* = 175)	2–5 years (*n* = 100)
**Site**	***n*** ** (%)**					
Be Part Research	343 (39.93%)	66 (37.08%)	87 (37.50%)	55 (31.61%)	85 (48.57%)	50 (50.00%)
Newtown Clinical Research Centre	343 (39.93%)	66 (37.08%)	87 (37.50%)	50 (28.74%)	90 (51.43%)	50 (50.00%)
Mecru Clinical Research Unit	173 (20.14%)	46 (25.84%)	58 (25.00%)	69 (39.66%)	0 (0.00%)	0 (0.00%)
**Sex**	**n (%)**					
Male	401 (46.68%)	58 (32.58%)	98 (42.24%)	97 (55.75%)	96 (54.86%)	52 (52.00%)
Female	458 (43.32%)	117 (65.73%)	134 (57.76%)	77 (44.25%)	79 (45.14%)	48 (48.00%)
**Race**	**n (%)**					
Black	838 (97.56%)	177 (99.44%)	228 (98.28%)	171 (98.28%)	166 (94.86%)	96 (96.00%)
Coloured	20 (2.33%)	1 (0.56%)	4 (1.72%)	3 (1.72%)	9 (5.14%)	3 (3.00%)
Indian/Asian	1 (0.12%)	0 (0.00%)	0 (0.00%)	0 (0.00%)	0 (0.00%)	1 (1.00%)
**Age (years)**						
Mean (SD)	19.52 (12.13)	33.96 (6.76)	27.62 (7.61)	14.17 (1.64)	8.50 (1.66)	3.68 (1.00)
Median	16.00	35.00	26.00	14.00	8.00	4.00
Min, Max	2.00, 45.00	19.00, 45.00	18.00, 44.00	12.00, 17.00	6.00, 11.00	2.00, 5.00
**BMI Index (kg/m** ^**2**^ **)**						
Mean (SD)	21.48 (6.43)	26.61 (6.05)	25.27 (6.29)	19.86 (3.52)	16.13 (2.12)	15.75 (1.27)
Median	19.70	25.70	23.30	19.25	16.00	15.80
Min, Max	10.30, 39.80	15.90, 39.70	16.30, 39.80	12.80, 37.70	11.50, 24.40	10.30, 18.30
**Viral Load**^‡^ **(copies/mL)**						
Mean (SD)	-	2427.94 (5956.16)	-	-	-	-
Median	-	201.00	-	-	-	-
**CD4 Count**^**†**^ **(cells/uL)**						
Mean (SD)	-	843.40 (342.75)	-	-	-	-
Median	-	792.50	-	-	-	-

*PLWH: People Living With HIV; BMI: body mass index; SBP: systolic blood pressure; DBP: diastolic blood pressure; SD: standard deviation

‡ PLWH who have a detectable Viral Load (*n* = 16)

† PLWH only (*n* = 178)

The overall study population was predominantly of Black race (97.6%). Males comprised 46.7% of the total study participants, with the proportion varying by group, 32.6% among PLWH, 42.2% among HIV-negative adults, and approximately ranging from 52% to 56% among younger cohorts.

Among the 178 PLWH, 162 participants (91.0%) had an undetectable viral load (< 40 copies/mL), whereas 16 (9.0%) exhibited detectable viremia (≥ 40 copies/mL). Observed values ranged from 55 to 24,000 copies/mL. The CD4 count values ranged from 241 to 1,937 cells/µL with mean and median 843.40 cells/µL (SD = 342.8) and 792.5 cells/µL, respectively. Assessment of the CD4 count showed that none of the participants met criteria for severe immunosuppression (< 200 cells/µL). Only 25 participants (14.0%) were categorized as having moderate immunosuppression (200–499 cells/µL), while the majority, 153 individuals (86.0%), demonstrated normal immune status (≥ 500 cells/µL).

### Anti-HEV IgG seroprevalence

Among the 859 participants who had anti-HEV IgG test results at baseline (before vaccination), 18.04% (155/859) were seropositive and 81.72% (702/859) were seronegative, whereas two participants had equivocal or borderline results for the ELISA test.

### Anti-HEV IgG seroprevalence by study site

**Fig. 2:**
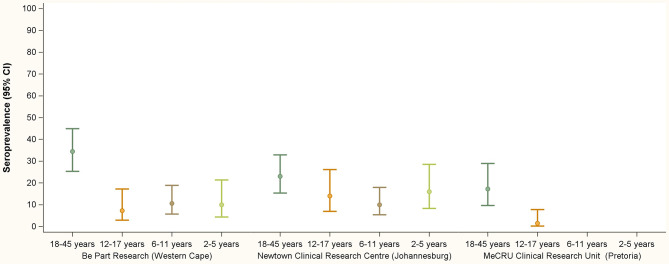
Anti-HEV IgG seroprevalence stratified by site and age group

Site-specific seroprevalence estimates were assessed by sex, age, and HIV status. Since the Mecru Clinical Research Unit site did not enroll children aged 2–5 years and 6–11 years, site comparisons were limited to adults and adolescents. Significant site-specific variation was observed among HIV-negative adults and adolescents, with higher seroprevalence at Be Part Research (34.5% and 7.3%) and Newtown Clinical Research Centre (23.0% and 14.0%) compared with Mecru Clinical Research Unit (17.2% and 1.5%, *p* = 0.0499 and 0.0262, respectively) (Table 2; Fig. 2).

**Table 2:** Anti-HEV IgG serostatus and seroprevalence by study site, sex, and age

Characteristics	Total			Be Part Research			Newtown Clinical Research Centre			Mecru Clinical Research Unit			*P*
	*N*	Seropositive, *n*	Seroprevalence (95% CI)	*N*	Seropositive, *n*	Seroprevalence (95% CI)	*N*	Seropositive, *n*	Seroprevalence (95% CI)	*N*	Seropositive, *n*	Seroprevalence (95% CI)	
**Sex**													
Male	401	66	16.46 (13.15, 20.40)	146	18	12.33 (7.94, 18.65)	165	38	23.03 (17.26, 30.02)	90	10	11.11 (6.15, 19.26)	**0.0119**[1]
Female	458	89	19.43 (16.07, 23.30)	197	48	24.37 (18.90, 30.81)	178	31	17.42 (12.55, 23.66)	83	10	12.05 (6.68, 20.78)	**0.0405**[1]
**HIV Status**													
18–45 years (PLWH)	178^*^	52	29.21 (23.03, 36.28)	66	18	27.27 (18.00, 39.04)	66	25	37.88 (27.15, 49.94)	46	9	19.57 (10.65, 33.17)	0.1009[1]
HIV-negative 18–45 years	232	60	25.86 (20.65, 31.86)	87	30	34.48 (25.34, 44.94)	87	20	22.99 (15.40, 32.86)	58	10	17.24 (9.64, 28.91)	**0.0499**[1]
**Age group**													
18–45 years	410	112	27.32 (23.23, 31.83)	153	48	31.37 (24.55, 39.10)	153	45	29.41 (22.77, 37.06)	104	19	18.27 (12.02, 26.78)	0.0525[1]
12–17 years	174	12	6.90 (3.99, 11.67)	55	4	7.27 (2.86, 17.26)	50	7	14.00 (6.95, 26.19)	69	1	1.45 (0.26, 7.76)	**0.0262**[2]
6–11 years	175	18	10.29 (6.61, 15.67)	85	9	10.59 (5.67, 18.91)	90	9	10.00 (5.35, 17.92)	-	-	-	0.8981[1]
2–5 years	100	13	13.00 (7.76, 20.98)	50	5	10.00 (4.35, 21.36)	50	8	16.00 (8.34, 28.51)	-	-	-	0.3724[1]

[Notes] seropositive rate was compared across sites using Chi-square test [1] or Fisher’s exact test [2]

*Two participants - one from Be Part Research and another from Newtown Clinical Research Centre, had equivocal or borderline anti-HEV IgG test results

### Anti-HEV IgG seroprevalence by age

Overall, the study findings demonstrate an age-related pattern in seroprevalence, highest in adults (25.9% in adults 18–45 years in the general population and 29.2% among PLWH adults), lower in young children (13.0% in those aged 2–5 years and 10.3% in those aged 6–11 years), and lowest among adolescents (6.9% in those aged 12–17 years).

Seroprevalence was markedly lower among children: 6.9% (95% CI: 4.0–11.7) in adolescents (12–17 years), 10.3% (95% CI: 6.6–15.7) in children aged 6–11 years, and 13.0% (95% CI: 7.8–21.0) in those aged 2–5 years (Table 2; Fig. 2). The age-stratified analysis also revealed significant site differences in adolescents aged 12–17 years (*p = 0.0262*). Newtown Clinical Research Centre had the highest seroprevalence in adolescents (14.0%), while Mecru Clinical Research Unit had the lowest (1.5%).

When all participants stratified by sex, females exhibited a slightly higher seroprevalence (19.4%, 95% CI: 16.1–23.3) compared to males (16.5%, 95% CI: 13.2–20.4). Seroprevalence differed significantly by sex across sites, where among males (*n* = 401), it ranged from 11.1% at Mecru Clinical Research Unit to 23.0% at Newtown Clinical Research Centre (*p = 0.0119*), and among females (*n* = 458), it ranged from 12.1% at Mecru Clinical Research Unit to 24.4% at Be Part Research (*p = 0.0405*) (Table 2).

**Fig. 3:**
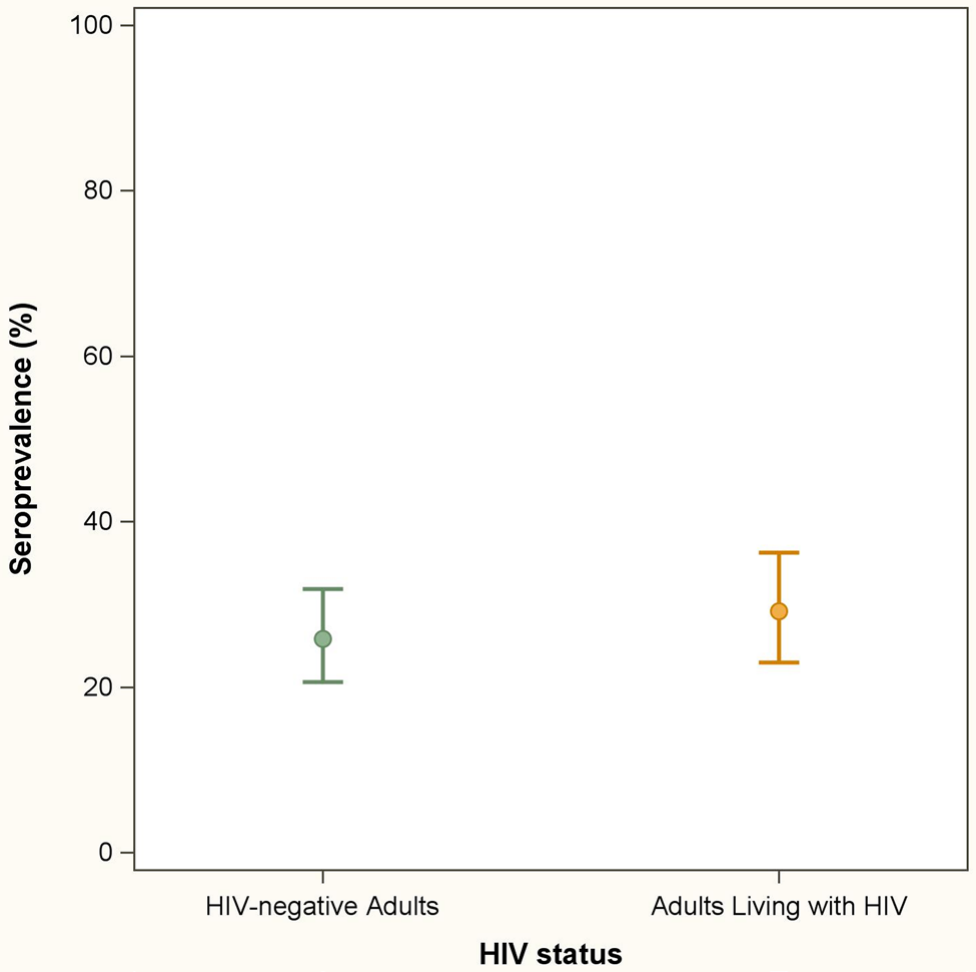
Anti-HEV IgG seroprevalence among adults stratified by their HIV status

Seroprevalence was also slightly higher among adult PLWH (29.2%; 95% CI: 23.0–36.3) than among HIV-negative adults (25.9%; 95% CI: 20.7–31.3), but the difference was not statistically significant (Table 2; Fig. 3). When stratified by site, adults in the general population (*n* = 232) had a statistically significant variation across sites (*p = 0.0499*), with the highest seroprevalence at Be Part Research (34.5%) and lowest at Mecru Clinical Research Unit (17.2%) (Table 2).

### Factors associated with anti-HEV IgG seropositivity

We assessed factors associated with anti-HEV IgG seropositivity among 859 participants, including age, sex, HIV status, virologic markers in PLWH, and site of enrolment (Table 3).

**Table 3:** Seroprevalence and crude odds ratios (ORs) with 95% confidence interval (CIs)

Variable	Seroprevalence % (95% CI)	Crude OR (95% CI)	*P*-value	aOR (95% CI)*	*P*-value
Sex					
Male	16.46 (13.15, 20.40)	0.82 (0.58, 1.16)	0.259	1.09 (0.75, 1.59)	0.653
Female	19.43 (16.07, 23.30)	1.0 (ref)	-	1.0 (ref)	-
Healthy Age group¥					
18–45 years	25.86 (20.65, 31.86)	2.33 (1.22, 4.48)	0.011	2.82 (1.45, 5.49)	**0.002**
12–17 years	6.90 (3.99, 11.67)	0.50 (0.22, 1.13)	0.096	0.66 (0.28, 1.52)	0.327
6–11 years	10.29 (6.61, 15.67)	0.77 (0.36, 1.64)	0.494	0.77 (0.36, 1.64)	0.497
2–5 years	13.00 (7.76, 20.98)	1.0 (ref)	-	1.0 (ref)	-
HIV Status in Adults					
HIV-negative	25.86 (20.65, 31.86)	0.85 (0.55, 1.31)	0.451	0.84 (0.54, 1.30)	0.433
PLWH^†^	29.21 (23.03, 36.28)	1.0 (ref)	-	1.0 (ref)	-
Virologic Markers in PLWH^‡^					
Viral Load					
Undetectable	30.25 (23.70, 37.71)	1.88 (0.51, 6.89)	0.341	1.88 (0.51, 7.00)	0.346
Detectable	18.75 (6.59, 43.01)	1.0 (ref)	-	1.0 (ref)	-
CD4 Count^§^					
SIM	-	-	-	-	-
MIS	24.00 (11.50, 43.43)	0.73 (0.28, 1.96)	0.538	0.68 (0.25, 1.83)	0.441
NIS	30.07 (23.36, 37.75)	1.0 (ref)	-	1.0 (ref)	-
Study Site					
Be Part Research	19.24 (15.42, 23.75)	1.82 (1.06, 3.12)	0.029	2.36 (1.36, 4.11)	**0.002**
Newtown Clinical Research Centre	20.12 (16.22, 24.68)	1.93 (1.13, 3.29)	0.016	2.64 (1.52, 4.59)	**0.001**
Mecru Clinical Research Unit	11.56 (7.61, 17.18)	1.0 (ref)	-	1.0 (ref)	-

* aOR: Adjusted ORs were derived from multivariable logistic regression model including age and site as covariates. When analyses were stratified by age group or site, the respective variable was omitted from the model. Reference categories are indicated by “ref”

¥ For age group comparisons, only children and HIV-negative/healthy adults were included, as HIV-positive adults were analyzed separately in comparison with HIV-negative adults

^†^ People Living With HIV

^‡^ PLWH only (*n* = 178)

^§^ SIM: Severe immunosuppression (< 200 cells/uL); MIS: Moderate immunosuppression (≥200 and < 500 cells/uL); NIS: Normal immune status (≥500 cells/uL)

Seroprevalence was slightly higher among females (19.4%) than males (16.5%), but there was no statistically significant association in either crude (OR 0.82, 95% CI: 0.58–1.16, *p* = 0.259) or adjusted analyses (aOR 1.09, 95% CI: 0.75–1.59, *p* = 0.653).

Compared with the reference group of children aged 2–5 years, adults aged 18–45 years had significantly higher odds of seropositivity (crude OR 2.33, 95% CI: 1.22–4.48, *p* = 0.011; aOR 2.82, 95% CI: 1.45–5.49, *p* = 0.002). Adolescents (12–17 years) and children aged 6–11 years did not show significant differences compared with the 2–5 years reference group.

Seroprevalence was slightly higher among PLWH adults (29.2%) compared with HIV-negative adults (25.9%), but the difference was not statistically significant (aOR 0.84, 95% CI: 0.54–1.30, *p* = 0.433). Virologic markers among PLWH: Adults with undetectable viral load had a higher seroprevalence (30.3%) than those with detectable viral load (18.8%), but the association was not statistically significant (aOR 1.88, 95% CI: 0.51–7.00, *p* = 0.346). CD4 count categories (SIM, MIS, NIS) were also not significantly associated with seropositivity.

Compared with Mecru Clinical Research Unit (reference, 11.6%), participants from Be Part Research (19.2%) and Newtown Clinical Research had significantly higher odds of seropositivity in both crude (Be Part Research OR 1.82, *p* = 0.029; Newtown Clinical Research Centre OR 1.93, *p* = 0.016) and adjusted analyses (Be Part Research aOR 2.36, *p* = 0.002; Newtown Clinical Research Centre aOR 2.64, *p* = 0.001).

### Anti-HEV IgG antibody titer

**Fig. 4:**
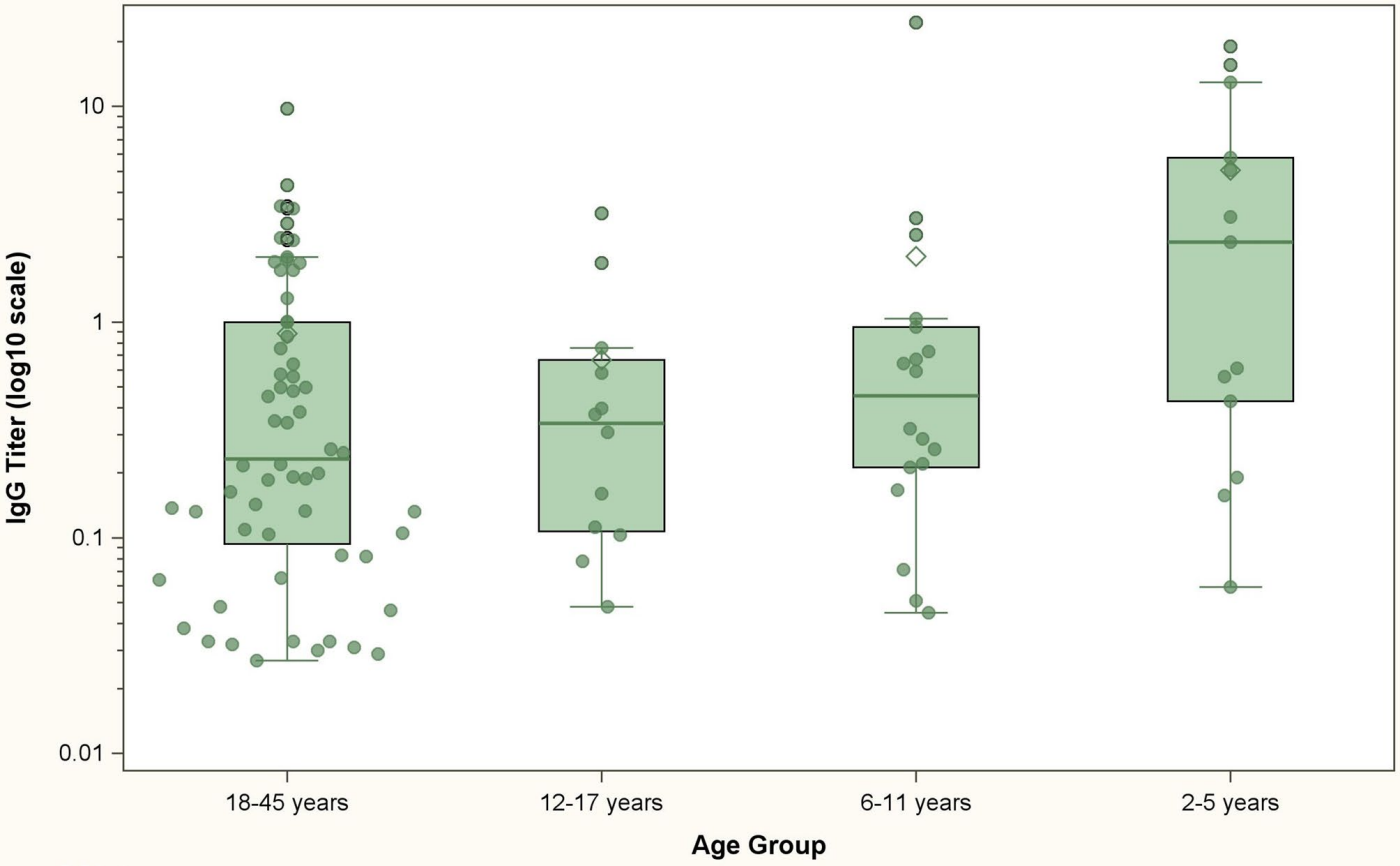
Baseline anti-HEV IgG antibody titers by age group among seropositive participants (18–45 years, *n* = 60; 12–17 years, *n* = 12; 6–11 years, *n* = 18; 2–5 years, *n* = 13)

Adult PLWH (*n* = 54) had a mean and a median anti-HEV IgG titer of 0.68 IU/mL (SD 0.96) and 0.24 (IQR 0.55), respectively, comparable to HIV-negative adults (*n* = 60) of the same age group (mean 0.88 IU/mL, SD 1.54 and median 0.29, IQR 0.91). Adolescents (*n* = 12) aged 12–17 years had a mean and median antibody titer of 0.67 IU/mL (SD 0.94) and 0.34 (IQR 0.56), respectively, while young children demonstrated higher titers, with mean and median levels of 2.02 IU/mL (SD 5.68) and 0.45 (IQR 0.74) in those aged 6–11 years (*n* = 18) and 5.06 IU/mL (SD 6.53) and 2.35 (IQR 5.35) in the 2–5-year group (*n* = 13), respectively (Fig. 4). The observed age-specific difference in anti-HEV IgG antibody titers was statistically significant (*p = 0.0371*).

## Discussion

In this study, we documented an overall anti-HEV IgG seroprevalence of 18.0% (95% CI: 15.6–20.8) among all participants comprising children, adolescents, and adults regardless of HIV status enrolled at three clinical sites in South Africa. Our findings reveal heterogeneity by age, HIV status, sex, and geographic location, underscoring a considerable variation in HEV epidemiology within South Africa. This moderate seroprevalence aligns with several African studies [27], but is lower than the highest reported rates on the continent including reports from South Africa [22–24], suggesting different factors play key roles in shaping HEV exposure patterns. Although this is a multisite study, the findings should be interpreted cautiously in the context of the study population, which consisted of volunteers enrolled in a vaccine clinical trial rather than participants selected through population-based sampling, as individuals in such studies may differ systematically from the broader population in terms of healthcare access, health-seeking behaviour, socioeconomic characteristics, and underlying health status, which may have influence on the observed seroprevalence patterns. Consequently, despite inclusion of multiple sites, the results may not be fully generalizable to the wider population at country or regional level.

The 18.0% (95% CI 15.6–20.8) overall estimated seroprevalence in our study is lower than the 27.9% (95% CI 25.3–30.5) reported from the Western Cape Province by Madden et al. (2016), despite both studies employed the same antibody detection assay, Wantai HEV IgG ELISA. Even when focusing specifically on our site located in Mbekweni, Western Cape Province, the seroprevalence estimate of 19.2% remains below the previously reported figure [22]. Differences in age structure, geographic context, and characteristics likely contribute to this discrepancy. The inclusion of a large paediatric group, who had the lowest seroprevalence, contrasts with the more adult-weighted population in the Western Cape study, where seropositivity rose sharply after adolescence (22).

We observed substantial geographic variation in anti-HEV IgG seroprevalence, with adult and adolescent participants from Johannesburg (29.4% and 14.0%) and Mbekweni, Western Cape (31.4% and 7.3%) showing higher seroprevalence compared to Garankuwa, Pretoria (18.3% and 1.5%, respectively). Similar variation has historically been documented in South Africa – early work demonstrated sporadic HEV circulation with higher seroprevalence in rural communities [21], while more recent reports have shown variation across provinces [23, 28]. A study among patients with acute hepatitis in Western Cape reported an anti-HEV IgG prevalence of approximately 29.5% [24], and the other study in the Western Cape on a comprehensive regional analysis confirmed an age-adjusted seroprevalence of 21.9% and identified pork consumption as a key exposure linked to zoonotic HEV genotype 3 transmission [22].

Environmental surveillance data from Salemane et al. (2024) identified HEV RNA in approximately 21.8% of wastewater and surface water samples across South African provinces, with particularly high detection in regions such as the Western Cape (38.1%) [29]. Historical seroepidemiologic data also showed high anti-HEV IgG seroprevalence (28% to 43%) in the Western Cape [24, 30]. In contrast, a seroprevalence study conducted near Pretoria found a low HEV antibody prevalence of 3.1% in HIV negative pregnant women, suggesting limited local HEV transmission [28]. These patterns may suggest that HEV exposure risk could be greater in Western Cape and Johannesburg compared to Pretoria.

In contrast, a 2024 study conducted in the Free State, South Africa found an exceptionally high IgG seroprevalence of 60.9%, suggesting a distinct epidemiological profile in that province [23]. Earlier serological study in specific South African populations demonstrated wide variability, with seroprevalence estimates influenced by local environmental and behavioural exposures [20]. Collectively, these findings highlight that HEV exposure in South Africa is not uniform but shaped by region-specific factors which mirrors global patterns of substantial heterogeneity driven by differences in sanitation, water quality, dietary practices, and animal reservoir distribution. These findings underscore the importance of multi-site sampling, regional risk assessment, and tailored public health strategies for accurately characterizing and addressing HEV epidemiology.

Overall, these findings highlight the value of multi-site sampling for estimating HEV epidemiology in South Africa, as exposure risk can vary by location – a pattern consistent with region-specific heterogeneity observed across Africa and globally. For example, in Uganda’s Rakai District, a community-based serosurvey found anti-HEV IgG seroprevalence of 47% among adults, signifying a very high burden of past HEV exposure in that rural setting [31]. A broader systematic review of HEV in Africa similarly documented variation in seroprevalence in different countries ranging from as low as 0% in some rural equatorial African villages residents in Gabon [27, 32] to extremely high rates of up to 84.3% in pregnant women in Egypt [27, 33]. Another meta-analysis focused on pregnant women across Africa estimated a pooled seroprevalence of 29.1% (95% CI 14.6–43.6%), with highest rates in North and East Africa and substantial heterogeneity by assay and geography [19]. Zoonotic contributions are also likely important: a systematic review of HEV in African animals reported a pooled IgG seroprevalence of 23.4%, with pigs showing particularly high rates (35.1%), underscoring the potential for cross-species transmission [34].

In this multi-site study, we also observed a clear age-dependent pattern in anti-HEV IgG seroprevalence, where adults had the highest seroprevalence, particularly PLWH (29.2%), followed by HIV-negative adults (25.9%), while children and adolescents had substantially lower seroprevalence rates. Our observations are partially consistent with those reported in the Western Cape Province of South Africa study, who also reported an increase in seroprevalence with age, from approximately 10% in children (< 19 years) to nearly 30% in adults aged 30–39 years and plateaued thereafter [22]. Another study from Western Cape also found a seroprevalence increase with age: 5% (≤ 21 years), 26% (22–45 years), and 46% (> 46 years) (*p* < 0.001) [35]. In Malawi, Taha et al. (2015) reported a 16.5% adult seroprevalence, indicating that exposure accumulates primarily in adulthood rather than early childhood [36]. The age-related seroprevalence pattern is even more pronounced in a Zambian study by Jacobs et al. who reported that seroprevalence rose sharply from 8% in children aged 1–4 years to 36% by ages 10–14, eventually reaching 42% in adults, with rates as high as 71% among PLWH [11]. Compared with Zambia, our study shows delayed acquisition of the HEV, with relatively lower seroprevalence in adolescents and a more pronounced rise only in adulthood.

However, recent longitudinal serological data from a population-representative cohort in Bangladesh suggest that one possible explanation for age-specific seroprevalence patterns is the waning of detectable antibodies over time. Over approximately 9 months of follow-up, about 15% of participants who were initially anti-HEV IgG positive lost detectable antibodies annually, and seroreversion was significantly more common in children than in adults. Most seropositive children below 10 years became seronegative within months, compared with much longer persistence in older age groups [37]. These findings raise the possibility that rapid loss of detectable antibodies during childhood could contribute to underestimation of prior exposure in cross-sectional studies and may result in an apparent trough in seroprevalence during adolescence, even if infections occurred earlier in life. Therefore, the low adolescent seroprevalence observed in our study may be consistent with antibody kinetics as well as differences in exposure patterns; however, these explanations cannot be distinguished using cross-sectional data alone and should be interpreted cautiously. Accounting for seroreversion is important because failure to consider antibody waning may lead to underestimation of infection risk when seroprevalence data are interpreted without information on antibody persistence.

The relatively low seroprevalence observed among adolescent participants (12–17 years) in our study may reflect several non-mutually exclusive explanations. One possibility is that detectable antibodies following earlier infection decline over time, while another is that exposure patterns differ across age groups. For example, adolescents may experience lower exposure than adults, who may have additional occupational or behavioural risk factors (e.g., animal contact or consumption of higher-risk foods) or effects arising from improvements in hygiene and food safety compared to young children. Because our study was cross-sectional, it is not possible to determine whether the observed age pattern reflects differences in exposure, antibody waning [37], cohort effects [38] or a combination of these factors.

In our study, the anti-HEV IgG seroprevalence was slightly higher in females (19.4%, 95% CI: 16.1–23.3) than in males (16.5%, 95% CI: 13.2–20.4), but this difference was not statistically significant (OR = 0.82, 95% CI: 0.75–1.59; *p* = 0.653) suggesting that sex is not a strong independent predictor of past HEV exposure in South Africa. This aligns with the previous studies done in South Africa: for instance, the recent study in the Free State province of South Africa, which reported more males (63.3%) than females (59.5%) were seropositive, but found no statistically significant difference in seropositivity by sex (*p* = 0.81) [23]. Similarly, the study from Western Cape of South Africa, reported no significant difference by sex [24]. However, studies in other settings and populations such as a study among PLWH in Central African Republic and a cohort study of PLWH in Nepal found significantly higher seroprevalence among women, suggesting that sex related differences in exposure risk may be more prominent in certain populations or socio-cultural settings [39].

Among our adult participants, PLWH exhibited a slightly higher anti-HEV IgG seroprevalence (29.2%, 95% CI: 23.0–36.3) than HIV-negative adults (25.9%, 95% CI: 20.7–31.9), but the difference was not statistically significant (OR = 0.85, 95% CI: 0.55–1.31; *p* = 0.451). Neither viral load nor CD4 count was significantly associated with HEV seropositivity, although these findings should be interpreted cautiously given the limited statistical power of some subgroup analyses. This is consistent with prior epidemiological evidence including a large meta-analysis of immuno-compromised populations including HIV-positive patients which found no significant difference in anti-HEV IgG seroprevalence between HIV-infected individuals and other immunosuppressed groups such as transplant recipients [40]. The cross-sectional study in the Rakai District, Uganda, involving 500 HIV-positive adults and matched HIV-negative individuals, observed a very high anti-HEV IgG seroprevalence (47%) but found no association between HIV status and anti-HEV IgG seropositivity (prevalence ratio 0.97, 95% CI: 0.85–1.11) [31]. However, in Zambia, a study found a strong association between HIV infection and HEV seroprevalence, where HIV was significantly associated with anti-HEV IgG positivity in adults, although the study population included individuals with very low CD4 counts [11]. The inconsistency of the association across settings and populations again suggests that HIV infection per se may not dramatically alter the likelihood of HEV exposure. However, these findings should be interpreted cautiously, as the HIV-positive adult participants were either on stable ART or required to have received the treatment for at least four weeks prior to enrolment, and were predominantly virologically suppressed with relatively preserved immune status.

Quantitative IgG measurements in our study provided important additional insight into the timing and intensity of HEV exposure across age groups and sites, where young children (2–5 years) had the statistically significant highest mean titres (5.06 IU/mL), whereas adults exhibited lower titres despite higher seroprevalence. This may suggest that younger children’s infections were more recent and that antibody levels may decline over time following exposure. This pattern is consistent with general seroepidemiologic understanding that antibody titers can reflect the recency and magnitude of exposure.

## Conclusions

In conclusion, our findings reveal a moderate overall anti-HEV IgG seroprevalence (approximately 18%), across these three geographic settings in South Africa. We observed an age-related seroprevalence pattern, characterized by the highest seroprevalence in adults, the lowest in adolescents, and comparatively high antibody titers in young children (2–5 years). These findings suggest recent exposure among those 2–5 years with declining seroprevalence and titers in later childhood and adolescence, followed by rising seroprevalence in adults possibly through cumulative new exposures. The lower prevalence observed among adolescents may represent a transitional phase with reduced exposure and/or waning antibody levels following early childhood infection, although this warrants further investigation. Seroprevalence also varied by site, suggesting that local environmental, sanitary, sociocultural or dietary factors may play an important role in shaping exposure risk. No significant association was observed between HEV seropositivity and HIV status, viral load, or CD4 count among adult PLWH on stable ART. These findings provide valuable insights into HEV epidemiology in South Africa and can inform vaccination and public health strategies, particularly in regions with heterogeneous exposure patterns.

### Strengths and limitations

Strengths of our study include a relatively large sample size drawn from multiple sites in South Africa with broad age group inclusion and including PLWH. In addition, this study includes quantitative assessment of anti-HEV IgG titers with all testing performed at a centralized lab using an international reference standard. Limitations include that the participants were not randomly selected from the communities but were volunteers in a clinical trial which may introduce selection bias. In addition, the cross-sectional nature of sampling cannot distinguish recent from past infections and no HEV genotyping or extended risk factor for exposure could be assessed beyond geographic residence, age, sex and HIV status.

## Data Availability

No datasets were generated or analysed during the current study.

## Abbreviations

aOR: Adjusted odds ratio
ANOVA: Analysis of variance
ARV: Antiretroviral
CD4: Cluster of Differentiation 4
CI: Confidence interval
ELISA: Enzyme-linked immunosorbent assay
HEV: Hepatitis E virus
HIV: Human immunodeficiency virus
IgG: Immunoglobulin G
IgM: Immunoglobulin M
IQR: Interquartile range
IU: International unit
IVI: International Vaccine Institute
OR: Odds ratio
Max: Maximum
MeCRU: Mecru Clinical Research Unit
MIS: Moderate immunosuppression
Min: Minimum
mL: Milliliter
NIBSC: National Institute for Biological Standards and Control
NIS: Normal immune status
PLWH: People living with HIV
SAHPRA: South African Health Products Regulatory Authority
SAS: Statistical Analysis System
SD: Standard deviation
SIM: Severe immunosuppression
SST: Serum separator tube
uL: Microliter
WHO: World Health Organization
